# Apatinib with EGFR-TKIs in advanced wild gene-type NSCLC: A case report

**DOI:** 10.1097/MD.0000000000013924

**Published:** 2019-01-04

**Authors:** Yuan-Peng Wu, Ji-Jin Wu, Shu-Mei Tian, Tao Jin, Chan Li, Ke Xie

**Affiliations:** a Department of Oncology, Xinjin County People's Hospital; b Department of Oncology, Sichuan Academy of Medical Sciences and Sichuan Provincial People's Hospital, Sichuan, P.R.China.

**Keywords:** apatinib, EGFR-TKIs, erlotinib, NSCLC

## Abstract

**Rationale:**

For advanced non-small-cell lung cancer (NSCLC), targeted therapy and chemoradiotherapy are recommended as the first-line treatment. For patients with a performance status (PS) score over 2 and without gene mutation, however, only supportive treatment is provided and survival time is extremely short. We believe that more can be done to improve the patient's survival time and their quality of life.

**Patient concerns and diagnoses:**

A 65-year-old female came to our hospital due to “cough and pain and lack of movement in the left leg”. The diagnosis was advanced wild gene-type lung adenocarcinoma and PS score over 2.

**Interventions and outcomes:**

She was treated in our clinic with apatinib and erlotinib and has had no progression of the disease for 15.4 months. Except for the presence of hand-foot syndrome and diarrhea, no other serious adverse reactions were seen.

**Lessons:**

For patients in poor physical condition and unacceptable of chemo-radiotherapy, apatinib combined with an epidermal growth factor receptor tyrosine kinase inhibitor (EGFR-TKI) is a safe and effective therapeutic method for advanced wild gene-type NCSCL.

## 1 Introduction

Lung cancer, which is one of the most malignant tumors, has the highest incidence among cancer patients in China. About 85% of patients present with non-small-cell lung cancer (NSCLC) and most are in an advanced stage.^[1]^ At present, the therapeutic methods include surgery, chemoradiotherapy, targeted therapy, anti-tumor neovascularisation, and immunotherapy. However, for patients without gene mutation and performance status (PS) scores over 2 points at a late stage, there are few options available.

Apatinib, a new kind of small-molecule tyrosine kinase inhibitor, can specifically bind to vascular endothelial growth factor receptor-2 (VEGFR-2). This binding inhibits the migration and proliferation of endothelial cells induced by VEGF thus reducing angiogenesis and micro-vessel density in the tumor.^[2,3]^ Apatinib has also proven to be safe and effective in the treatment of advanced gastric cancer.^[4]^ The application of the drug in treating many solid tumors such as hepatocarcinoma, breast cancer, lung cancer, and esophageal carcinoma is being explored.^[5]^

Erlotinib, a first generation epidermal growth factor receptor tyrosine kinase inhibitor (EGFR-TKI) drug, blocks the activity of EGFR tyrosine kinase, thus inhibiting the activation of downstream pathways and reducing angiogenesis and the proliferation, differentiation, and metastasis of tumor cells. Administering erlotinib provides better therapeutic benefits than standard chemotherapy for EGFR-mutated NSCLC.^[6]^ It was approved by the Food and Drug Administration (FDA) as early as 2013, but only for EGFR-mutated NSCLC. Since then, 1 published trial has demonstrated that NSCLC patients that have the wild-type form of EGFR still benefit from EGFR-TKI.^[7]^

## 2 Case report

A 65-year-old female came to our hospital on May 6, 2016 due to “cough and pain and lack of movement in the left leg”. The diagnosis was adenocarcinoma of the left upper lobe of the lung with involvement of both lungs, mediastinal lymph nodes, liver, and multiple bone metastases (cT4N2M1b, stage IV).Since the biopsy specimens were too small, a liquid biopsy was carried out and no gene mutation was found. According to the National Comprehensive Cancer Network (NCCN) Treatment Summary for NSCLC, anti-angiogenesis drugs combined with chemotherapy was recommended for advanced wild gene-type NSCLC when the PS score was 0 to 2. The PS of this patient at the time of diagnosis was 2 to 3 points which precluded the use of chemotherapy and apatinib was recommended. Because of the potential of liquid biopsies to give false negative results and because the patient belonged to the high-risk EGFR mutation group, she was also administered erlotinib with monthly infusions of zoledronic acid to prevent bone destruction and accelerate bone regeneration.

In further evaluating the patient, we found no active bleeding, no enterobrosis, no ileus, no cardiac insufficiency, and no anaphylaxis. Her hepatorenal function was normal and her hypertension was controlled. After signing a consent form on May 26, 2016, 250 mg po qd of apatinib (supported by Heng Rui Pharmaceutical Co., Ltd.) was given with a 28-day cycle. On July 2, 2017, 150 mg po qd erlotinib was administered. Due to severe diarrhea, the erlotinib dose was reduced to 75 mg qd 1 month later. After treatment, the pain in the left leg was significantly improved and she was able to walk normally.

**Figure 1:**
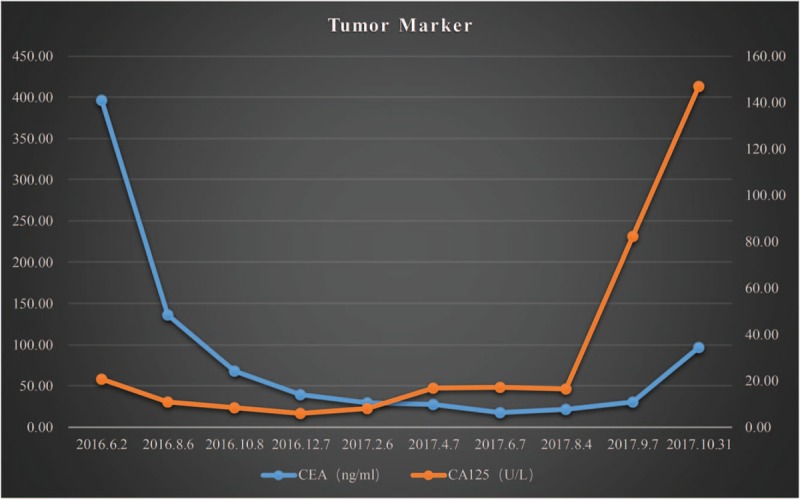
Levels of CEA and CA125 throughout therapy. CA125 = OC125 antigen, CEA = carcinoembryonic antigen.

**Figure 2:**
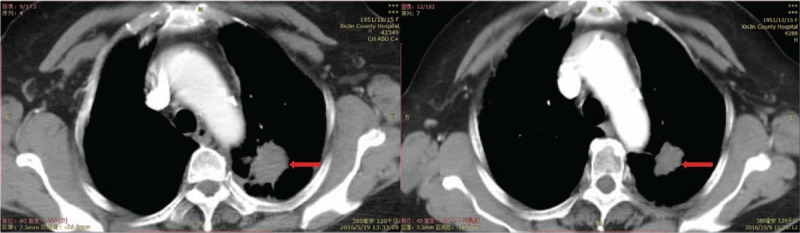
Computed tomography shows the mass in the left lung on mediastinal window before and after treatment.

**Figure 3:**
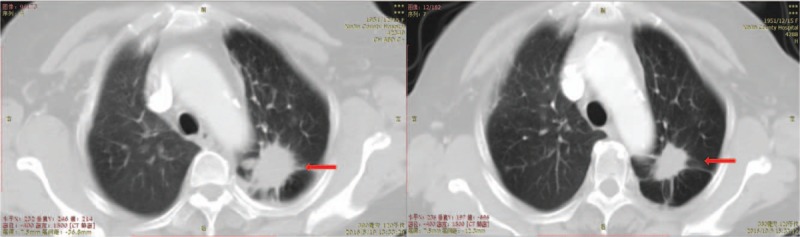
Computed tomography shows the mass in the left lung on lung window before and after treatment.

**Figure 4:**
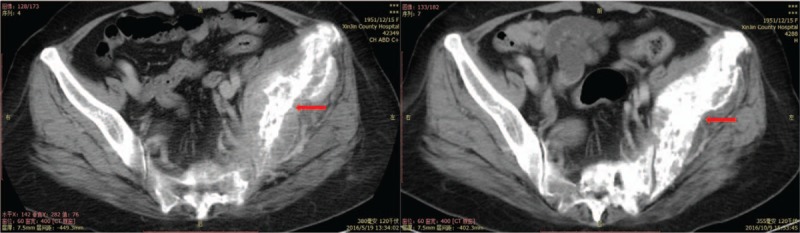
Computed tomography shows the mass in the left pelvis before and after treatment.

**Figure 5:**
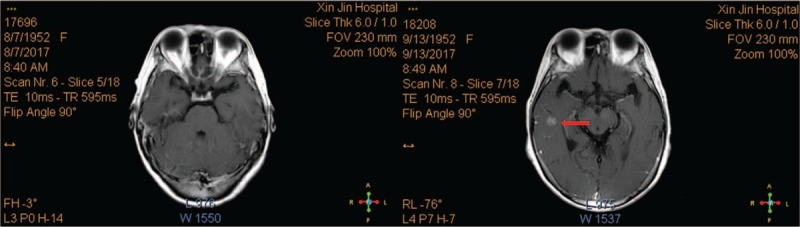
Magnetic resonance imaging shows the nodules in the brain before and after metastasis.

**Figure 6:**
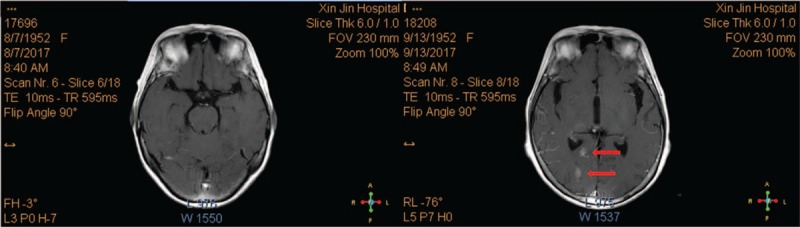
Magnetic resonance imaging shows the nodules in the brain before and after metastasis.

The tumor markers, carcinoembryonic antigen (CEA), and OC125 antigen (CA125) were rechecked and levels had dropped from 396.4 ng/mL to 67.90 ng/mL and from 20.63 U/mL to 8.28 U/mL respectively (Fig. 1). Her computed tomography (CT) showed that the displacement of the left lung was significantly smaller than before (Figs. 2 and 3) and that the left iliac bone mass was reduced (Fig. 4). Subsequently, the partial response (PR) efficacy was rechecked regularly and stable disease (SD) condition evaluated. The patient was hospitalized again for fainting and urinary incontinence on September 7, 2017. We found that the tumor marker levels had increased (Fig. 1), and an intracranial magnetic resonance imaging (MRI) showed multiple abnormally strengthened intracranial nodular shadows, suggesting metastasis (Figs. 5 and 6). In view of the apparent tumor progression, it was proposed to repeat the genetic test again. We still had to use a liquid biopsy because of the difficulty of sampling and the results again showed no gene mutation. Local radiotherapy was recommended but the patients and her family refused, opting instead for continuing with the self-treatment of apatinib combined with erlotinib.

**Figure 7:**
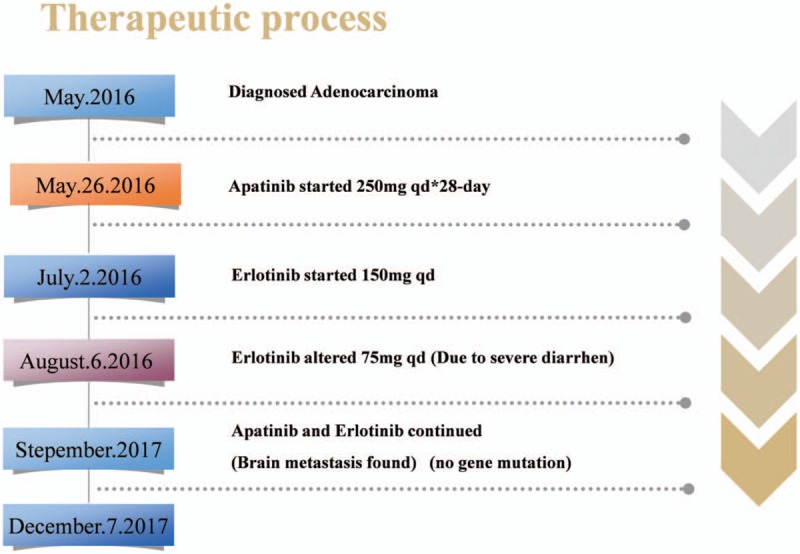
The whole therapeutic process including apatinib and erlotinib, start time, dose titration, and treatment after progressive disease.

In December 2017, the patient came to our hospital again for fainting. An MRI showed that the number of intracranial nodules was slightly increased and they were larger than before. On December 7, 2017, the patient agreed to radiotherapy and the Gamma Knife was adopted for treatment. Her current condition is stable. The entire therapy regimen is shown in Figure 7.

## 3 Discussion

VEGF and VEGFR are the most important regulatory factors for tumor angiogenesis, and by specifically inhibiting their activity, tumor angiogenesis can be reduced so as to promote tumor necrosis.^[8]^ As a broad-spectrum anti-VEGF monoclonal antibody, bevacizumab has been proven safe and effective in non-squamous NSCLC. Better outcomes have been achieved, however, when bevacizumab was combined with chemotherapy and targeted therapy,^[9]^ which has been approved as a first-line combined chemotherapy and targeted therapy regimen. Through competitive inhibition of VEGFR-2, apatinib blocks the combination between VEGF and VEGF-2. This inhibits VEGFR-2 phosphorylation, down-regulates phosphorylation of its downstream extracellular signal-regulated kinase, and blocks tumor angiogenesis. Currently, phase II clinical trials on advanced gastric cancer,^[10]^ advanced breast cancer^[11]^ and recurrent epithelial ovarian cancer^[12]^ have preliminarily confirmed the safety and efficacy of apatinib, and a phase III clinical trial on gastric cancer further confirmed its safety and efficacy.^[13]^

Around 2 weeks after beginning apatinib treatment, the patient presented with hand-foot syndrome and mucosal reaction. The main manifestations were skin cracks and damage to the soles of both feet and mild diarrhea. There was no evidence of active whole body hemorrhage, liver damage, proteinuria, or weakness.

Mutations in the EGFR gene are the most common mutations seen in NSCLC. By competitively binding with the adenosine triphosphate (ATP) sites on intracellular receptors and inducing the proliferation of homodimers or heterodimers, EGFR-TKIs block the activity of EGFR kinase. This inhibits the activation of downstream pathways, thus reducing proliferation, differentiation, angiogenesis, and metastasis of tumor cells. The efficacy of EGFR-TKIs has been confirmed and they are approved as a first-line treatment for NSCLC.^[14]^ A subtype analysis showed that EGFR mutations were more pronounced in Asians, females, non-smokers, and patients with adenocarcinoma of the lungs.^[15]^ The patient reported on here belongs to the high-risk EGFR mutation group. We used the liquid biopsy method to detect mutations, which has a sensitivity of about 85%,^[16]^ but a relatively large probability of false negatives. In consequence of this and with the goal of blocking tumor neovascularization, we opted to use EGFR-TKIs. After a month of erlotinib treatments, the patient was having diarrhea with a frequency of 5 to 6 times daily. The diarrhea was not completely relieved by loperamide hydrochloride, so we reduced the erlotinib dose to 75 mg po qd.

Research has shown that resistance will inevitably appear under treatment with EGFR-TKIs. Domestic and foreign studies have confirmed that after 9 to 13 months of first-line treatment with EGFR-TKI, the vast majority of patients showed disease progression.^[6,17]^ The T790 mutation is the most common mechanism for EGFR-TKI to acquire drug resistance. Studies showed that in NSCLC without EGFR-TKI, the incidence of T790 mutation was less than 0.1%, while 50% of patients receiving EGFR-TKI acquired resistance resulting from T790 mutations.^[18]^

After the disease progressed, our patient again underwent comprehensive mutation detection screening including 20p.T790.No mutations were found, which further proves that the patient indeed had a wild gene-type NSCLC. Of course, this can’t exclude the existence of other extremely rare mutations that have not yet been detected. When the first generation of EGFR-TKIs is found to become ineffective through the development of resistance, the second-generation afatinib,^[19]^ or the third generations AZD9291^[20]^ are potential alternatives. At the least, we can hope that these newer drugs will delay the acquisition of drug resistance and prolong survival. Peng YM et al^[21]^ have reported that adding apatinib to the treatment after development of EGFR-TKI resistance, can add 5.1 months of PFS (progression-free survival), but the mechanism of action remains unknown. In the case reported here, we found that apatinib combined with erlotinib increased the patient's PFS by 15.4 months. The patient's survival time was significantly prolonged and her quality of life was improved.

## 4 Conclusion

For patients in poor physical condition and unacceptable of chemo-radiotherapy, apatinib combined with an EGFR-TKI is a safe and effective therapeutic method for advanced wild gene-type NCSCL. Additional clinical trials are needed to substantiate this result, however.

## Acknowledgments

The authors thank Dr. Gary Bentley of on the Mark scientific editing service for English language editing and helpful comments.

## Author contributions

All authors contributed toward data analysis, drafting and revising the paper and agreed to be accountable for all aspects of the work.

**Formal analysis:** Yuan-Peng Wu.

**Methodology:** Chan Li.

**Project administration:** Shu-Mei Tian, Tao Jin.

**Software:** Ji-Jin Wu.

**Writing – original draft:** Yuan-Peng Wu, Ji-Jin Wu.

**Writing – review & editing:** Ke Xie.
